# One’s Workplace, Other’s Home? Work and Health of Domestic Workers in Argentina

**DOI:** 10.29024/aogh.2311

**Published:** 2018-08-31

**Authors:** María Fernanda Bauleo, Frank van Dijk, Katja Radon

**Affiliations:** 1 MunBaus, Av. Mariano Acosta 1254, (1407), Buenos Aires, AR; 2 Center for International Health LMU @ Institute and Outpatient Clinic for Occupational, Social and Environmental Medicine, University Hospital of Munich (LMU), Ziemssenstr. 1, D-80336 Munich, DE

## Abstract

**Background:**

Domestic workers around the world work and eventually live in private homes where control of working conditions is difficult.

**Objective:**

The aim of this study was to compare working conditions and its impact on general and mental health in live-in and live-out domestic workers in Argentina.

**Methods:**

In a cross-sectional study, the Spanish version of the European Workings Condition Survey and an ad hoc questionnaire were applied to 201 domestic workers (response 94%). Twelve months’ prevalence of verbal or physical workplace violence was assessed. Poor general health was defined by general health self-assessed as poor or fair. Symptoms of common mental disorders (CMD) were considered present if Goldberg’s general health score was above 4. Data were analyzed using Chi square’s test and logistic regression models.

**Findings:**

Live-in workers formed 66% of the participants. They were more likely to take care of the elderly, iron and cook than live-out workers. Workplace violence was reported by 17% of live-in and 24% of live-out workers (p = 0.25). Overall prevalence of poor general health was 23%; 53% reported CMD. After adjustment, violence remained a statistically significant predictor of poor general health (Odds Ratio 7.3; 95% Confidence Interval 2.8–19.1) and CMD (3.2; 1.1–9.3).

**Conclusions:**

Working conditions of live-in and live-out domestic workers are different. However, exposure to workplace violence is common in both groups and affects general and mental health.

## Introduction

Nowadays, a majority of middle or upper-middle class homes function thanks to domestic workers, who do domestic duties while the householders are working in an income-generating activity [1]. Outsourcing household work [2] and eventually care of children or the elderly is usually a woman-woman relationship based on the traditional gender division of labor [3,4]. According to the International Labor Organization (ILO), almost 53 million workers are employed in paid domestic work in low-, middle- and high-income countries, the majority of them women [5]. In Latin America, one in six women in the labor market is a domestic worker [4]. In Argentina, at least 803.436 female workers were in this job in 2012, 17% of them coming from bordering countries or from Peru. In Buenos Aires, about 40% of domestic workers are migrants [6].

In words of the ILO, “domestic work is performed in or for one or more households” whilst a “domestic worker” is a “person engaged in domestic work within an employment relationship” [7]. In this sense, the Argentinean domestic service law considers “domestic work” as all work taking place in the private households, implying all services or executions of cleaning tasks, maintenance or other traditional household activities. Personal assistance, children care, as well as non-therapeutic care of sick or disabled people are included [8]. This law also establishes labor rights such as overtime payment; sick and maternity leave; annual vacation; and social security (health care service and pension). The domestic workers could do their job living out (live-out) or in (live-in) the employer’s home. In the case of live-in workers, the employer has to provide them with a private furnished room. They have the right to three resting hours between morning and afternoon, nine sleeping hours and a weekly rest of at least 35 continuous hours. Meanwhile, the live-out workers may work at maximum 9 hours a day for 5 days a week.

Nevertheless, these legal requirements are not always fulfilled as outside control is limited and the fact that the domestic employee depends on the employer – even more in case of illegal migrants [8,9]. Domestic work is invisible in society [10,11], and many employers are not aware that domestic labor might have negative impacts on health. However, intimacy with the employer [12], a variety of psychosocial [13], physical, biological and chemical [11] conditions were shown to be important risk factors for the health and safety of domestic workers [14]. A live-in regimen could mean more working hours, lack of intimacy, social isolation and little rest [5,15], but also more social support by the employing family [12,16,17]. To our knowledge, no study so far investigated the working conditions and health of domestic workers living in the employers’ home compared to live-out workers. The jobs’ informality and the fact that the home is the workplace make it challenging to investigate this population [18]. To our knowledge, no data for Argentina exist.

The aim of this study was thus to compare psychosocial working conditions as well as general and mental health in live-in and live-out domestic workers in Argentina.

## Materials and Methods

### Study Population

The field work was carried out in December 2010/January 2011 and December 2012/January 2013. In a cluster sampling approach, five employment agencies located in the city of Buenos Aires that publish advertisements in the local press were invited to participate in the survey. These agencies are private and specialized in household jobs. Three of them refused participation because they were concerned about the results of the study and their potential implication for their business. The two remaining agencies distributed the self-administered questionnaires to all women working in domestic labor. Women received written information about the study objectives, the voluntary character of the survey and the anonymity of the responses. Oral informed consent was sought. Of the 217 eligible women, 201 (93.7%) completed the survey. After completion, questionnaires were disposed in a sealed box collected by the principal investigator (MFB). The 16 non-responders were either afraid of not being employed through the agency if they answered the questionnaire, mistrusted the survey or – in one case – did not complete the survey because the woman was unable to read.

The Ethics Committees of the Institute of Occupational, Social and Environmental Medicine of the Munich University, Germany, and the Professional Board of Chemical Engineering in Buenos Aires, Argentina approved this study.

### Study Instruments and Variable Definition

The self-administered questionnaire contained items of the “Encuesta Nacional de Condiciones de Trabajo” (National Working Conditions Survey, derived from the European Working Condition Survey) [19] and an ad hoc questionnaire, developed and expert validated for the population under study. Items included sociodemographics, employment conditions, working conditions, job tasks, experience of verbal or physical violence at work, demand-control perception, social support, physical health and mental health.

Based on the questionnaire response, participants were divided into two groups defining those who reported that they live in the employers’ home as live-in and those who live outside the employer’s home as live-out domestic workers.

Sociodemographic variables considered were age (less than 29 years; 30–39 years and ≥40 years), level of education (primary education or less, uncompleted secondary education, at least secondary education), nationality (migrant yes/no) and family group (living alone, living as a couple, living with children, living with a relative).

Work-related variables included years of service divided by the median of the distribution (<4 years, 4–38 years), domestic tasks (child care, care of elderly, ironing, cooking, cleaning), and type of employment (formal vs. informal).

Job demand (four items: working at high speed, working to tight deadlines, not having enough time to get the job done and pace of work depending on work of others), job autonomy (seven items on design-, activity- and decision-latitudes), and social support (three items on possibility to get assistance and having good relationships at work) were assessed on 5-point Likert scales from 1 = never to 5 = always [20]. Each item was transformed by [21].

x = 1 + \frac{{{\rm{response\,\,on\,\,Likert\,\,scale}} - 1}}{{{\rm{Number\,\,of\,\,response\,\,categories}} - 1}}

The resulting transformed items of each scale were summed up and dichotomized at their median.

Verbal violence was considered present if participants reported any communication problems, personal discredit or threats at work 12 months prior to survey. Physical violence was defined as any violence from family members, other persons at the workplace or sexual harassment at work during the 12 months prior to survey. The overall 12-month prevalence of violence was calculated based on presence of either verbal or physical violence. Missing data in these variables were considered as presence of violence. In sensitivity analyses, we excluded missing data on violence.

Two outcomes were considered. The screening question “How is your health in general?” taken from the EWCS and SF-36 [22,23], was used as physical health marker [24]. Poor health was considered present when this item was self-rated as poor or fair [24]. Symptoms of common mental disorders (CMD) [25] were evaluated by the Goldberg Questionnaire GHQ-12 [26,27]. The 0-0-1-1 scaling method was used resulting in a scale from 0–12. A cutoff of >4 was used to define poor mental health [28].

### Statistical Analysis

For double data entry (with error check) and statistical analyses, we used EpiInfo version 3.5.4 [29]. Two women were excluded from the analyses as they did not indicate whether they lived in or out of the employer’s house.

To test for independence between live-in and live-out domestic workers, sociodemographic factors, work tasks, psychosocial working conditions and work-related violence were compared using Chi^2^-test. Thereafter, prevalence of poor general health and CMD was calculated for each of these variables. Ironing (93%), and cleaning (98%) were not considered in these analyses as they were carried out by almost all participants. Finally, Odds Ratios (ORs) with 95% Confidence Intervals (95% CI) were calculated by logistic regression models. Risk factors associated with the outcomes at p_Chi_^2^ < 0.05 were included in the final adjusted model. Crude and mutually adjusted ORs were compared. Missing data on items included in the adjusted model were excluded from the crude and adjusted logistic regression models.

## Results

### Descriptive results

Of the 199 participants, two women reported currently not working due to an occupational accident, another two due to an occupational disease and one was currently on vacation. The study population was young, with 45% being under 30 years and 15% 50 years or older (latter data not shown). More than 50% had only a primary education. About 65% of the participants were migrants, of whom 86% were from Paraguay while the remaining came from Peru (8%), Bolivia (3%), Uruguay (1.5%) and the Dominican Republic (1.5%). Live-in and live-out workers did not differ statistically significantly on sociodemographic parameters (Table 1).

**Table1:** Sociodemographic characteristics of 199 female domestic workers from Buenos Aires, Argentina, stratified for living in or living out of employer’s home.

Characteristics		Total	Living In	Living Out	N_Missing_	p_Chi_^2^

		% (n)	% (n)			

**Participants**		100 (201)	65.8 (131)	34.2 (68)	0	
**Age (years)**	≤ 29	45.3 (91)	45.8 (60)	42.6 (29)	0	0.33
	30–39	25.9 (52)	22.9 (30)	32.4 (22)		
	≥40	28.9 (58)	31.3 (41)	25.0 (17)		
**Education**	≤Primary	52.8 (103)	51.9 (67)	54.5 (36)	0	0.72
Incomplete secondary		16.4 (32)	15.5 (20)	18.2 (12)		
≥Secondary completed/superior		30.8 (60)	32.6 (42)	27.3 (18)		
**Migrant**	No	35.2 (70)	34.4 (45)	36.8 (25)	0	0.74
	Yes	64.8 (129)	65.6 (86)	63.2 (43)		

The main work tasks were child care, cleaning, cooking and ironing. Live-in domestic workers were more likely to take care of elderly (48% vs. 29%; p = 0.01), to cook (93% vs. 79%; p = 0.004) and to iron (95% vs. 87%; p = 0.03) than those living outside the employer’s home. Job demand, job control and social support were lower among live-in workers than live-out workers; however, differences reached only the level of statistical significance for social support (38% vs. 57%; p = 0.02). Almost 20% of the study population reported having experienced any kind of violence at the workplace during the 12 months prior to the survey with no statistically significant differences between live-in (17%) and live-out (24%) workers (p = 0.25; Table 2).

**Table 2:** Working conditions of 199 female domestic workers from Buenos Aires, Argentina, stratified for living in or living out of employer’s home.

Variable		Living in N = 131	Living out N = 68	N_Missing_	p_Chi_^2^

		% (n)	% (n)		

**Duration of employment (years)**	<4	51.9 (67)	46.2 (30)	5	0.45
	4–38	48.1 (62)	53.8 (35)		
**Work tasks**					

**Child care**	Yes	81.7 (107)	79.4 (54)	0	0.70
**Care of elderly**	Yes	48.1 (63)	29.4 (20)		0.01
**Ironing**	Yes	95.4 (125)	86.8 (59)		0.03
**Cooking**	Yes	93.1 (122)	79.4 (54)		0.004
**Cleaning**	Yes	98.5 (129)	97.1(66)		0.5
**Psychosocial working conditions**					

**Job demand score**	>Median	43.7 (55)	46.3 (31)	6	0.73
**Job control score**	>Median	46.0 (57)	56.3 (57)	11	0.18
**Social support score**	>Median	37.5 (45)	56.5 (35)	17	0.02
**Physical or verbal workplace violence**	Yes	16.8 (22)	23.5 (16)	0	0.25

The overall prevalence of poor general health was 23%, again without statistically significant difference between live-in (27%) and live-out (16%) workers (p = 0.09). Shorter duration of employment, child care, job demand above the median of the distribution and violence were the main predictors of poor general health in the bivariate analyses (Table 3). After mutual adjustment, living in the employer’s house was associated with an increased odds of poor general health (Odds Ratio 2.9; 95% Confidence Interval 1.2–7.2). Duration of employment (4–38 years compared to <4 years: 0.4; 0.2–1.0), child care (6.1; 1.2–30.8) and violence (7.3; 2.8–19.1) were also associated with the outcome (Table 4).

**Table 3:** Prevalence of poor general health and common mental disorders by sociodemographic factors and working conditions of 199 female domestic workers from Buenos Aires, Argentina.

Variable		Poor general health^1^ N = 199^3^		CMD^2^ N = 186^3^	

		% (n)	p_Chi_^2^	% (n)	p_Chi_^2^

**Overall prevalence**		23.1 (46)	N/A^4^	53.2 (99)	N/A^4^
**Age (years)**	≤29	25.8 (23)	0.12	41.2 (35)	<0.001
	30–39	28.8 (15)		76.5 (39)	
	≥40	13.8 (8)	3	50.0 (25)	
**Education**	≤Primary	28.2 (29)	0.09	43.8 (42)	0.005
	Incomplete secondary	27.3 (9)		75.8 (25)	
	≥Secondary completed/superior	13.6 (8)		56.6 (30)	
**Migrant**	No	22.9 (16)	0.95	55.4 (36)	0.67
	Yes	23.3 (30)		52.1 (63)	
**Living-in**	No	16.2 (11)	0.09	62.7 (37)	0.08
	Yes	26.7 (35)		48.8 (62)	
**Duration of employment (years)**	<4	30.9 (30)	0.01	37.6 (35)	<0.001
	4–38	15.5 (15)		68.2 (60)	
**Child care**	No	5.3 (2)	0.004	66.7 (24)	0.07
	Yes	27.3 (44)		50.0 (75)	
**Care of elderly**	No	21.6 (25)	0.54	54.7 (58)	0.64
	Yes	25.3 (21)		51.2 (41)	
**Cooking**	No	13.0 (3)	0.22	75.0 (15)	0.04
	Yes	24.4 (43)		50.6 (84)	
**Job demand score**	≤Median	17.8 (19)	0.04	45.4 (44)	0.06
	>Median	30.2 (26)		59.5 (50)	
**Job control score**	≤Median	27.4 (26)	0.14	59.8 (52)	0.11
	>Median	18.3 (17)		47.8 (43)	
**Social support score**	≤Median	25.5 (26)	0.20	46.9 (45)	0.24
	>Median	17.5 (14)		55.8 (43)	
**Physical or verbal workplace violence**	No	17.4 (28)	<0.001	48.1 (74)	0.002
	Yes	47.4 (18)		78.1 (25)	

^1^ Self-reported general health poor or fair. ^2^ Symptoms of common mental disorders (GHQ-12 > 4). ^3^ N_missing_ see tables 1 and 2. ^4^ NA Not applicable.

**Table 4:** Bi- and multivariable associations between potential sociodemographic and work-related risk factors and poor general or mental health. Results of the crude and adjusted logistic regression models including all variables with p < 0.05 in the bivariate analyses (Table 3).

Variable		Poor general health^1^ N = 188		CMD^2^ N = 178	

		cOR (95% CI)^3^	aOR (95% CI)^4^	cOR (95% CI)^3^	aOR (95% CI)^4^

**Age (years)**	≤29	–	–	1	1
	30–39	–	–	4.8 (2.1–10.7)	3.3 (1.2–8.0)
	≥40	–	–	1.5 (0.7–3.0)	0.9 (0.4–2.2)
**Education**	≤Primary	–	–	1	1
	Incomplete secondary	–	–	3.8 (1.5–9.3)	3.3 (1.2–9.0)
	≥Secondary completed/superior	–	–	1.8 (0.9–3.6)	1.9 (0.9–4.0)
**Living-in**	No	1	1	1	1
	Yes	2.0 (0.9–4.5)	2.9 (1.2–7.2)	0.6 (0.2–1.2)	0.7 (0.3–1.4)
**Duration of employment (years)**	<4	1	1	1	1
	4–38	0.5 (0.2–0.9)	0.4 (0.2–1.0)	3.8 (2.0–7.0)	2.5 (1.1–5.4)
**Child care**	No	1	1	–	–
	Yes	6.2 (1.4–27.2)	6.1 (1.2–30.8)	–	–
**Cooking**	No	–	–	1	1
	Yes	–	–	0.3 (0.1–1.0)	0.4 (0.1–1.4)
**Job demand score**	≤Median	1	1	–	–
	>Median	2.0 (1.0–3.9)	1.7 (0.8–3.7)	–	–
**Physical or verbal workplace violence**	No	1	1	1	1
	Yes	4.7 (2.1–10.4)	7.3 (2.8–19.1)	4.0 (1.5–10.4)	3.2 (1.1–9.3)

^1^ Self-reported general health poor or fair. ^2^ Symptoms of common mental disorders (GHQ-12 > 4). ^3^ Crude Odds Ratio with 95% Confidence Interval. ^4^ Adjusted Odds Ratio with 95% Confidence Interval.

Over 50% of the population (53%) reported GHQ-12 scores above 4 indicating CMD. Age, higher level of education, longer duration of employment, cooking as a work task and experience of violence at the workplace were statistically significantly associated with CMD in the bivariate analyses (Table 3). Prevalence of CMD was not statistically significantly lower for live-in (49%) than live-out domestic workers (63%; p = 0.08). After mutual adjustment, age between 30 and 39 years (OR 3.3; 95% CI 1.2–8.0), incomplete secondary education (3.3; 1.2–9.0), duration of employment of four years or more (2.5; 1.1–5.4) were risk factors for CMD. Living in employers home was not associated with this outcome (0.7; 0.3–1.4). As for general health, experience of violence at the workplace increased the odds of CMD (3.2; 1.1–9.3).

In the sensitivity analyses, leaving out missing data on violence from the analyses did not change the association with general health (OR 11.4; 95% CI 3.1–41.3). For CMD, multivariable modelling was not possible as all those who had experienced violence also reported CMD.

## Discussion

This cross-sectional study in 201 domestic employees working in Buenos Aires indicates a very high prevalence of poor self-perceived general as well as mental health. In addition, it shows differences in work tasks and social support between live-in and live-out domestic employees. General health was worse in female domestic workers living in their employer’s house, taking care of children and suffering from violence at the workplace. The latter exposure was also an important risk factor for CMD.

The few existing previous studies indicated that living in or out of the employer’s home results in important differences in work environment and working conditions [30,31,32]. In general, recently arrived immigrants prefer being a live-in worker to safe money for housing and to live under better housing conditions [15]. In our study population, the prevalence of migrants was high in live-in (66%) and live-out (63%) workers. The lack of difference might be because many migrants in our study population have lived in Argentina for a long time; we did not assess how long migrants had lived in Argentina.

Our findings indicate that live-in domestic workers are more likely to take care of the elderly, being caregivers and, at the same time, domestic workers. The increasing demand of live-in domestic staff for this task is in line with other studies [32] due to the increasing age of populations [33] in many countries and the low costs of a domestic worker as compared to a nursing home [30,31,34]. In our study, taking care of the elderly was not associated with health. Recent studies indicate that caregivers’ general and mental health is strongly dependent of individual motivation and skills as well as on specific situations in the household they live in [32,35,36]. This might explain the lack of association seen in our study. Furthermore, domestic workers in our study living in the employer’s house were more likely to iron and cook. The larger variety of tasks is most likely related to the fact that live-in domestic employees may work more hours a week than those not living in their employers’ homes.

The SF-36 was validated in the adult clinical population of a large university hospital in Buenos Aires [37]. In this study [37], the overall prevalence of poor or fair self-rated general health was 7.5% as compared to 23.1% in our study population. No prevalences were given stratified for gender, age or level of education [37]. Unfortunately, we did not assess the type of the health problems our study population was suffering from. Comparing the GHQ-12 results of our study population to results of a recent validation study in Cordoba, Argentina, the same picture can be seen: while the prevalence of CMD was 53% in domestic workers, it was 28% in the female population of Argentinian primary care centers (28%) [28]. One has to take into account that our study population came from a metropolitan city were the prevalence of CMD might be somewhat higher than in a smaller city like Cordoba. In addition, our study population was slightly older; however, in the validation study no age trend was seen. Nevertheless, described differences in general health and CMD between our study population and the general Argentinian population are so large that one may assume that they are not due to confounding but could at least partly be related to working conditions, migration status and income level.

The main predictors of poor general health and CMD was experience of verbal or physical violence at the workplace. About one in five women reported such experiences in the 12 months prior to the study. This might indicate the vulnerability of the job inside the employer’s house without outside control or other type of protection of the workers. Only a few studies assessing the prevalence of violence against domestic workers were found. One study indicated a 12-month prevalence of sexual harassment of 27% among domestic workers in Sao Paulo, Brazil [38] – a prevalence much higher than in our study (3%, data not shown). However, comparison is limited as definitions of violence differed. Nevertheless, as in our study, exposure was related to poor mental health. Looking at cleaners in general, a Peruvian study indicated a 12-month prevalence of 39% physical violence in hospital cleaners compared to 8% in non-cleaners [39]. All this supports that cleaners are a vulnerable group for exposure to violence. The situation is not only limited to low- and middle-income countries: a recent descriptive report draws attention to violence especially against migrant domestic workers in the UK [40].

The demand-control-social support model is a well-known predictor of general and mental health [41,42,43]. In our study, live-in domestic workers were less likely to receive social support at the workplace than live-out workers. One may hypothesize that being with the employer’s family around-the-clock makes one feel less supported than only spending some hours per day at the workplace. This is in contrast to previous studies indicating higher social support by the employing family in the case of live-in workers [12,16,17]. Likewise, we did not see the previously described association between social support and the health outcomes [42,44]. Nevertheless, we saw a tendency for an association between higher job demand and poor general health supporting the demand-control [25,42].

Several limitations of our study should be mentioned. The small sample size resulted in limited statistical power. Therefore, some confidence intervals were large. In addition, this might explain why e.g., job demand lost statistical significance in the adjusted model. Our study population were women currently seeking new employment. One may speculate that one reason for looking for a new job could be discontent with the previous one. Another potential source of selection bias is that only some of the agencies invited to participate in the study agreed to do so. The main reason for this was that participation meant extra work for the agencies without direct benefit. As the agencies do not have direct influence or control over working conditions, we do not think that the participation in the survey was related to working conditions or health of the domestic workers. In addition, we did not assess the level of Spanish of the participants. All women in our study population came from Spanish-speaking neighbouring countries in which local languages (e.g., Guaraní) are also used. However, as the agencies perform the job interviews in Spanish and our questionnaire was only provided in Spanish, our study population might not be representative for non-Spanish-speaking migrants from these countries.

The inverse association between duration of employment and poor general health as well as the decreasing association age and CMD after age 40 years indicates a healthy worker survivor effect [45]. We assumed that missing information of workplace violence was not at random but that women hesitated to report violence. Therefore, we counted women not answering the items on violence as being exposed. Our sensitivity analyses confirm this assumption, as results only marginally changed for general health while no single victim of violence without CMD was found. Assessing exposure and outcome by questionnaire might result in common methods bias and thus in spurious findings. The low level of education of half of the study population might have resulted in problems understanding the instrument, however, we expect the resulting misclassification of exposure and outcomes to be non-differential. Finally, general health was based on only one generic self-reported answer. However, this item is a validated standard measure to assess general health [22,37]. Our study population only contained female domestic employees working in Buenos Aires. The results therefore cannot be generalized to other countries or to male domestic workers. In addition, the fact that women were recruited via employment offices may reduce the ability to generalize, as many domestic workers are directly employed. One may hypothesize that women employed officially through an office work under better conditions; however, we cannot prove this assumption. Among the strengths of this research are its high response, the use of validated questionnaire instruments and the low item non-response. Furthermore, a large variety of psychological working conditions and work tasks were assessed, and two health outcomes were included.

For policy and practice, we recommend a full compliance with the law, improving the domestic workers’ work environment and reducing informal employment. Nevertheless, because the domestic employee’s workplace is a private household, it is not easy to organize governmental audit, so the employer-employee working relationship has no direct witnesses. Accordingly, we suggest more cohort and qualitative studies in a large and varied population of domestic workers to distinguish occupational risks and their consequences on health, in order to develop informational material for employers and employees, and special worker trainings. When it could be possible, such activities should be part of a program involving syndicates and other organizations that can intervene in vulnerable situations.

In conclusion, our findings confirm a very high prevalence of poor general health and CMD among female domestic workers in Buenos Aires. Working conditions differ substantially between live-in and live-out workers, with living in the employer’s house being a predictor of poor general health. Verbal and physical violence is common. Better control of private households as working places is warranted. As this is difficult to accomplish, domestic workers should be empowered.
